# Shiga Toxin–Producing *Escherichia coli* O157:H7 Illness Outbreak Associated with Untreated, Pressurized, Municipal Irrigation Water — Utah, 2023

**DOI:** 10.15585/mmwr.mm7318a1

**Published:** 2024-05-09

**Authors:** BreAnne Osborn, Jennifer Hatfield, William Lanier, Jennifer Wagner, Kelly Oakeson, Ravyn Casey, Jacob Bullough, Pallavi Kache, Shanna Miko, Jasen Kunz, Grace Pederson, Molly Leeper, Nancy Strockbine, Haley McKeel, Jessica Hofstetter, Alexis Roundtree, Amy Kahler, Mia Mattioli

**Affiliations:** ^1^Utah Department of Health and Human Services; ^2^Utah County Health Department, Provo, Utah; ^3^Career Epidemiology Field Officer Program, CDC; ^4^Epidemic Intelligence Service, CDC; ^5^Division of Foodborne, Waterborne, and Environmental Diseases, National Center for Emerging and Zoonotic Infectious Diseases, CDC.

**Summary**

**What is already known about this topic?**

Municipal irrigation water systems are underrecognized possible sources of waterborne illnesses.

**What is added by this report?**

In 2023, at least 13 children in Utah became ill during an outbreak of Shiga toxin–producing *Escherichia coli* O157:H7 associated with untreated, pressurized, municipal irrigation water. Seven children were hospitalized, including two with hemolytic uremic syndrome. Nearly all children (12 of 13) reported using this untreated water for unintended purposes, including recreation and drinking.

**What are the implications for public health practice?**

Educating residents of communities with these irrigation systems about the risks of playing in or drinking untreated water and improving management and operations risk mitigation of these untreated water systems could help prevent the occurrence of waterborne illness outbreaks.

## Abstract

During July–September 2023, an outbreak of Shiga toxin–producing *Escherichia coli* O157:H7 illness among children in city A, Utah, caused 13 confirmed illnesses; seven patients were hospitalized, including two with hemolytic uremic syndrome. Local, state, and federal public health partners investigating the outbreak linked the illnesses to untreated, pressurized, municipal irrigation water (UPMIW) exposure in city A; 12 of 13 ill children reported playing in or drinking UPMIW. Clinical isolates were genetically highly related to one another and to environmental isolates from multiple locations within city A’s UPMIW system. Microbial source tracking, a method to indicate possible contamination sources, identified birds and ruminants as potential sources of fecal contamination of UPMIW. Public health and city A officials issued multiple press releases regarding the outbreak reminding residents that UPMIW is not intended for drinking or recreation. Public education and UPMIW management and operations interventions, including assessing and mitigating potential contamination sources, covering UPMIW sources and reservoirs, indicating UPMIW lines and spigots with a designated color, and providing conspicuous signage to communicate risk and intended use might help prevent future UPMIW-associated illnesses.

## Investigation and Results

### Identification of the Outbreak and Characteristics of Cases

Shiga toxin–producing *Escherichia coli* (STEC) O157:H7 is an enteric illness that can cause hemolytic uremic syndrome (HUS), a severe, life-threatening condition which affects the kidneys; young children (aged <5 years) are among the most susceptible to HUS. During July 25–30, 2023, six cases of STEC O157:H7 illness in children were reported to the Utah County Health Department (UCHD), with onset during July 22–27. All six ill children lived in city A, Utah. UCHD investigators interviewed the identified children’s parents using standard case investigation forms to assess various exposures before illness onset. Preliminary whole genome sequencing (WGS) found that two ill children’s clinical isolates were zero alleles different from each other, suggesting that an outbreak was occurring. On July 31, an outbreak investigation was initiated. 

**FIGURE 1:**
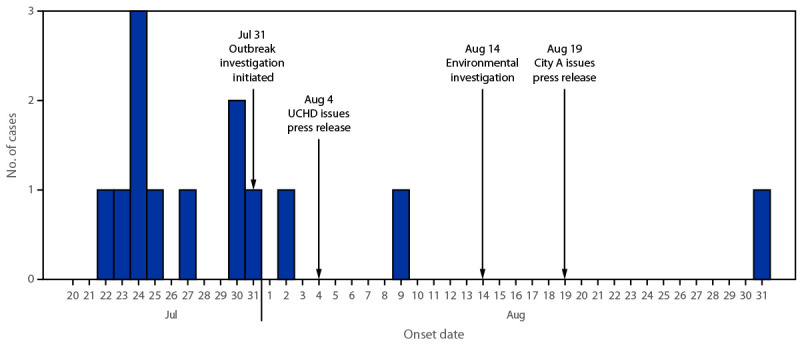
Cases of reported Shiga toxin–producing *Escherichia coli* O157:H7 illnesses, by onset date* (N = 13) — city A, Utah, July–August 2023 **Abbreviation**: UCHD = Utah County Health Department. * At the time of the August 4 press release, only eight cases had been reported to public health officials.

Investigators identified 13 children with confirmed STEC O157:H7 illness linked to this outbreak, with illness onsets during July 22–August 31 (Figure 1). The median patient age was 4 years (range = 1–15 years). Seven patients were hospitalized, including two with HUS; no deaths were reported. This activity was reviewed by CDC, deemed not research, and was conducted consistent with applicable federal law and CDC policy.*

### Association of Outbreak with Exposure to Untreated, Pressurized, Municipal Irrigation Water

Investigators developed a questionnaire to obtain additional exposure information for these cases and those that were later identified, including details regarding exposure to untreated, pressurized, municipal irrigation water (UPMIW), which had frequently been reported in preliminary interviews. UPMIW is surface water piped from reservoirs to homes; it is intended for outdoor landscapes (lawns and gardens) and is not suitable for drinking or recreational activities. UPMIW is not routinely monitored or tested.

All city A residences and businesses have outdoor connections to the UPMIW system. City A’s UPMIW is primarily sourced from mountain snow melt and is carried >30 miles (>48 km) by a river and an underground pipeline to several open UPMIW reservoirs within city A and surrounding communities before being pumped to residential connections. Secondary sources of city A’s UPMIW system comprise public wells and natural surface waters, including nearby rivers and creeks.

Twelve of 13 ill persons reported UPMIW exposure in city A during the week before symptom onset, including playing with hose water (five), inflatable lawn water toys (three), and water tables (two); drinking (two); and running through sprinklers (one). Among seven ill persons with discrete UPMIW exposure dates, the median incubation period was 3 days (range = 1–5 days). The one ill person who did not report UPMIW exposure was not a city A resident but did report spending time in city A during the week preceding symptom onset. No ill persons are known to have eaten noncommercial produce irrigated with UPMIW.

### Environmental Investigation

On August 14, investigators conducted an environmental investigation at two of city A’s UPMIW reservoirs and nine sites where persons with confirmed illness reported UPMIW exposure, including private homes. Investigators collected large-volume water samples by dead-end ultrafiltration and grab samples (unfiltered water collected in 1 liter bottles according to Environmental Protection Agency and CDC protocols^†^); samples of sediment and bird feces from the reservoirs; and swabs of spigots, hoses, toys, and other surfaces likely to have had contact with UPMIW. Investigators observed birds on and around UPMIW reservoirs during the environmental investigation; no other animals or obvious potential sources of STEC O157:H7 were observed during sampling.

### Laboratory Investigation

Investigators submitted samples of bird feces to the Utah Public Health Laboratory for STEC O157:H7 culture and submitted all other environmental samples to CDC for culture of STEC O157:H7 (*1*–*3*), followed by WGS of confirmed STEC O157:H7 isolates. Grab water samples were tested for generic *E. coli* and total coliforms per 100 mL. Microbial source tracking was performed for all dead-end ultrafiltration samples and both reservoir sediment samples (*4*–*6*). Microbial source tracking is used to detect microbial markers specific to the feces of avian species, ruminants (such as cattle, sheep, and deer), and humans (all known fecal shedders of STEC).^§^ Clinical laboratories submitted stool samples from ill persons to the Utah Public Health Laboratory for STEC O157:H7 culture, isolation, and WGS.

STEC O157:H7 was isolated from UPMIW reservoir sediment and dead-end ultrafiltration water samples from five of nine exposure sites. STEC O157:H7 was not detected in any other environmental samples collected.

**FIGURE 2:**
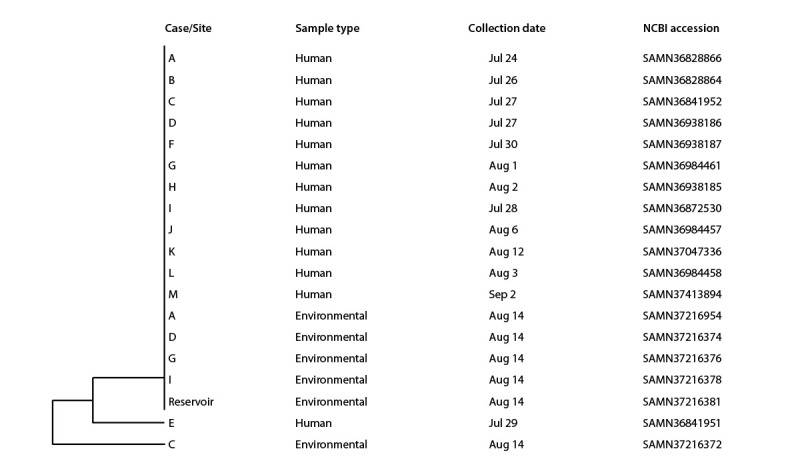
Genetic relatedness* of clinical and environmental isolates from samples collected during the outbreak investigation — city A, Utah, July–August 2023 **Abbreviation:** NCBI = National Center for Biotechnology Information. * Isolates from cases A–M and environmental samples A, D, G, and I and from the reservoir differ from case E and environmental sample C by a median of zero alleles (range = 0–1) and a median of zero alleles (range = 0–2), respectively, by core genome multilocus sequence typing.

WGS results indicated clinical isolates were within 0–1 allele difference of each other, and the environmental isolates were all within 0–2 allele differences of the clinical isolates by core genome multilocus sequence typing (Figure 2).^¶^ All sequencing analysis was conducted using the WGS analysis software platform (version 7.6; BioNumerics). Results from generic *E*. *coli* and total coliform testing performed on water grab samples were variable (Table), and two exposure sites with detectable STEC O157:H7 had no detectable coliforms or generic *E. coli* (<1 most probable number per 100 mL). Of 12 samples analyzed by microbial source tracking (10 dead-end ultrafiltration and two sediment), avian, ruminant, and human fecal markers were detected in 10, six, and one dead-end ultrafiltration samples, respectively, and the avian marker was detected in both sediment samples.

**TABLE:** Environmental testing results of a Shiga toxin–producing *Escherichia coli* O157:H7 outbreak investigation — city A, Utah, July–August 2023

Site*	Grab water samples (MPN/100 mL)		Dead-end ultrafiltration water samples	
	Total coliforms^†^	*E*. *coli*^†^	Microbial source tracking^§^	*E. coli* O157:H7
Exposure site A	<1	<1	Avian: detected	Detected
			Ruminant: ND	
			Human: ND	
Exposure site C	344.8	5.1	Avian: detected	Detected
			Ruminant: detected	
			Human: ND	
Exposure site D	866.4	8.5	Avian: detected	Detected
			Ruminant: ND	
			Human: ND	
Exposure sites E and F	5.2	<1	Avian: detected	ND
			Ruminant: ND	
			Human: ND	
Exposure site G	NC	NC	Avian: detected	Detected
			Ruminant: detected	
			Human: detected	
Exposures site H	686.7	3.1	Avian: detected	ND
			Ruminant: detected	
			Human: ND	
Exposure site I	<1	<1	Avian: detected	Detected
			Ruminant: detected	
			Human: ND	
Exposure site J	285.1	4.1	Avian: detected	ND
			Ruminant: detected	
			Human: ND	
Exposure site K	228.2	10.8	Avian: detected	ND
			Ruminant: detected	
			Human: ND	
City A UPMIW reservoir	Grab #1: 33.1	Grab #1: 2.0	Avian: detected	ND
	Grab #2: 613.1	Grab #2: 9.6	Ruminant: ND	
			Human: ND	
City A UPMIW reservoir sediment #1	NA	NA	Avian: detected	ND
			Ruminant: ND	
			Human: ND	
City A UPMIW reservoir sediment #2	NA	NA	Avian: detected	Detected
			Ruminant: ND	
			Human: ND	

**Abbreviations:** *E. coli* = *Escherichia coli*; MPN = most probable number; NA = not applicable; NC = not collected; ND = not detected; UPMIW = untreated, pressurized, municipal irrigation water.

* Exposure sites A–K were named based on the location of each ill person’s reported UPMIW exposure.

^†^ Total coliforms and *E*. *coli* (fecal indicator bacteria) were measured using Environmental Protection Agency standard methods, IDEXX Colilert-18 or Enterolert, on 100-mL grab water samples paired with dead-end ultrafiltration water samples. Sediment was not tested for total coliforms and *E*. *coli*.

^§^ Microbial source tracking assays were conducted using methods described in https://journals.asm.org/doi/10.1128/aem.04137-13, https://academic.oup.com/jambio/article/108/3/974/6720002?login=true, and https://www.frontiersin.org/articles/10.3389/fsufs.2019.00124/full.

^¶^ Detection of *E*. *coli* O157:H7 in environmental samples was determined using methods from https://assets.publishing.service.gov.uk/media/5be99537ed915d6a203046fd/SCA_Blue_Book_236.pdf, https://assets.publishing.service.gov.uk/media/5be9964e40f0b667b363e25d/MoDWPart4-223MAYh.pdf, and https://pubmed.ncbi.nlm.nih.gov/19363065/.

** A grab water sample was not collected at exposure site G.

## Public Health Response

UCHD issued a press release on August 4 (after identification of the first eight cases), before the environmental investigation, notifying the public of the outbreak, and warning against drinking or playing in UPMIW. After the press release, two additional cases were reported. On August 19, city A issued a second press release, stating that STEC O157:H7 had been detected in UPMIW samples and recommending that residents cook homegrown produce and avoid watering lawns and renewed warnings not to drink or play in UPMIW. City A distributed mailers on August 28, further informing residents of the risks associated with using UPMIW for drinking or recreation. City A is also assessing other prevention strategies, including water treatment and reservoir cleaning.

The Utah Department of Health and Human Services issued a Health Alert Network message about the outbreak on August 22, encouraging health care providers to perform stool testing for persons with diarrheal illness, educating providers about signs and symptoms of HUS, and warning against antibiotic treatment for STEC infections because treatment might increase the risk for HUS (*7*).

## Discussion

UPMIW systems are generally uncommon in the United States; however, they are used in some Utah communities to irrigate residential outdoor landscapes. These systems were designed to conserve drinking water and reduce water treatment costs. Utah UPMIW systems are not intended for drinking or recreation, are not monitored or tested for water quality, and, except for 2022 state legislation requiring metering,** are not currently regulated by state or local authorities.

City A installed an upgraded drinking water system in 1989 and, subsequently, established its UPMIW system by converting its previous drinking water system to a UPMIW system. Because UPMIW is also used by city A for fire suppression, it remains available to residents year-round, although its use is only encouraged during landscape irrigation season, usually mid-April through mid-October.

Epidemiologic and laboratory evidence confirmed UPMIW as the vehicle of this community STEC O157:H7 outbreak. In 2010 and 2015, two other Utah cities experienced campylobacteriosis outbreaks that were suspected to have been caused by cross-connections between UPMIW and drinking water lines (Utah Department of Health and Human Services, unpublished data, 2010 and 2015). Data from city A’s outbreak did not specifically implicate homegrown produce as an illness-causing vehicle, but previous outbreaks demonstrated that produce grown with water containing STEC O157:H7 can cause illness (*8*,*9*). Additional data are needed to understand risks associated with consuming noncommercial produce irrigated with UPMIW.

Notably, two exposure sites (A and I) where STEC O157:H7 was detected had undetectable levels of generic *E*. *coli* and total coliforms. Similarly, STEC O157:H7 was detected in produce irrigation water with low generic *E*. *coli* and total coliform levels during an investigation into a 2018 multistate outbreak associated with romaine lettuce.^††^ This finding is not surprising, given that generic *E. coli* testing cannot detect STEC O157:H7 (*10*). Thus, this testing, although widely used as an indicator of water quality, is not a reliable indicator of the presence of STEC O157:H7.

Although UPMIW is not intended for recreation, all but one child with UPMIW exposure in this outbreak reported some kind of play in the water. Utah water providers have previously instructed residents to not drink or play in UPMIW; however, recent population growth within city A might have resulted in residents who arrived more recently being uninformed about UPMIW-associated risks. This outbreak demonstrates the need for ongoing educational efforts and reminders. Educating residents of communities with UPMIW systems, especially those at higher risk for severe illness (including older adults, children, and persons with compromised immune systems) about the importance of using UPMIW for its intended purposes as well as the risks associated with drinking and recreational exposure, could prevent future cases of UPMIW-associated waterborne illness. In addition, water utilities could assess UPMIW systems for potential contamination sources and consider risk mitigation interventions, including covering UPMIW sources and reservoirs, more prominent labeling of UPMIW at public sites, distributing conspicuous signage for residents to use in their yards, and color coding UPMIW spigots and lines, as is recommended for other nonpotable water sources to prevent the occurrence of waterborne illness.^§§^

## Footnotes

* 45 C.F.R. part 46.102(l)(2), 21 C.F.R. part 56; 42 U.S.C. Sect. 241(d); 5 U.S.C. Sect. 552a; 44 U.S.C. Sect. 3501 et seq.

†
https://cfpub.epa.gov/si/si_public_record_Report.cfm?dirEntryId=355726&Lab=CESER

§
https://cfpub.epa.gov/si/si_public_record_Report.cfm?Lab=NRMRL&dirEntryID=133523

¶
https://www.applied-maths.com/bionumerics

**
https://le.utah.gov/~2022/bills/static/HB0242.html#73-10-34.5

††
https://www.fda.gov/media/117512/download?attachment

§§
https://www.epa.gov/sites/default/files/2019-08/documents/2012-guidelines-water-reuse.pdf
